# Home Pulse Rate Before and During Antihypertensive Treatment and Mortality Risk in Hypertensive Patients: A Post Hoc Analysis of the HOMED‐BP Study

**DOI:** 10.1161/JAHA.124.037292

**Published:** 2024-12-14

**Authors:** Takahiro Kimura, Masahiro Kikuya, Kei Asayama, Yukako Tatsumi, Yutaka Imai, Takayoshi Ohkubo

**Affiliations:** ^1^ Department of Hygiene and Public Health Teikyo University School of Medicine Tokyo Japan; ^2^ Fourth Department of Internal Medicine Mizonokuchi Hospital, Teikyo University School of Medicine Kawasaki Japan; ^3^ Department of Preventive Medicine and Epidemiology Tohoku Medical Megabank Organization, Tohoku University Sendai Japan; ^4^ Environment and Health KU Leuven Department of Public Health and Primary Care Leuven Belgium; ^5^ Tohoku Institute for Management of Blood Pressure Sendai Japan

**Keywords:** cardiovascular disease, cohort study, heart rate, home blood pressure, neoplasm, self‐measured, Epidemiology, Lifestyle, Cardiovascular Disease, Risk Factors, Hypertension

## Abstract

**Background:**

Although a high pulse rate assessed in the clinic office setting has been associated with an increased risk of cardiovascular disease and mortality, there are few studies assessing the prognostic ability of out‐of‐office pulse rate, particularly self‐measured home pulse rate.

**Methods and Results:**

We investigated the prognostic ability of home pulse rate in 3022 patients with mild‐to‐moderate hypertension. During a median follow‐up of 7.3 years, 72 patients died and 50 had major adverse cardiovascular events. For each 1 SD increase in pulse rate before treatment (9.4 beats per minute), the adjusted hazard ratio for all‐cause mortality was 1.52 (95% CI, 1.24–1.92). For each 1 SD increase in pulse rate during the follow‐up period (9.9 beats per minute), the adjusted hazard ratio was 1.70 (95% CI, 1.39–2.08). However, pulse rate was not significantly associated with major adverse cardiovascular events. When both home pulse rate and office pulse rate before treatment were included in a Cox model, only the home pulse rate significantly predicted all‐cause mortality (*P* ≤0.019). Excluding the home pulse rate from the model led to a significant deterioration of the model fit statistic (*P* ≤0.020). The optimal cut‐off values of home pulse rate in predicting all‐cause mortality, determined by Youden's index from a receiver operator characteristic analysis, were 67.8 beats per minute at baseline and 66.4 beats per minute during follow‐up.

**Conclusions:**

In patients with mild‐to‐moderate hypertension, the pulse rate measured at home, both before and during antihypertensive treatment, was associated with mortality risk and has superior prognostic ability compared with office pulse rate. The accuracy of risk stratification may be improved by using a home pulse rate, which can be self‐measured easily and frequently at home.

**Registration:**

URL: https://www.umin.ac.jp/ctr; Unique identifier: C000000137.

**Nonstandard Abbreviations and Acronyms**
HOMED‐BP: Hypertension Objective Treatment Based on Measurement by Electrical Devices of Blood Pressure Study
MACE: major adverse cardiovascular events

**Clinical Perspective**

**What Is New?**

Our study revealed an association between home pulse rate, both before and during antihypertensive treatment, and the risk of all‐cause mortality.

**What Are the Clinical Implications?**

The home pulse rate is a valuable predictor, and the accuracy of risk stratification may be enhanced by utilizing a home pulse rate, which can be easily and frequently self‐measured at home.

Known studies^1^, ^2^, ^3^, ^4^, ^5^, ^6^, ^7^ have reported a positive association of pulse rate measured during office visits and at health checkups, and mortality^1^, ^2^, ^3^, ^4^, ^5^, ^7^ and cardiovascular events^5^, ^6^ among patients with hypertension^1^, ^7^ and the general population.^2^, ^3^, ^4^, ^5^, ^6^ A consensus statement by the European Society of Hypertension^8^ and the Japanese Society of Hypertension Guidelines for the Management of Hypertension (JSH 2019)^9^ recommend routine measurement of office pulse rate for patients with hypertension. However, an optimal pulse rate is not defined since optimal rate and improvement of prognosis in individuals by controlling pulse rate are still under debate.^9^

Out‐of‐office blood pressure (BP) has more reproducibility, reliability, and better predictive ability than office BP and is widely recommended in clinical practice.^9^, ^10^, ^11^ Although similar advantages could be assumed for the out‐of‐office pulse rate,^8^ there is even less evidence on the prognosis of the out‐of‐office pulse rate,^12^, ^13^, ^14^, ^15^ and most studies have focused on pulse rate measured with ambulatory BP monitoring.^12^, ^13^, ^14^

Unlike ambulatory pulse rate monitoring, home pulse rate measurement is characterized by the advantages of measuring under standardized measurement methods and conditions.^9^, ^16^ Furthermore, ≈40% of Japanese people measure their pulse rate at home using home BP devices.^17^ It could be clinically meaningful to investigate the prognostic significance of home pulse rate; however, limited evidence is available.^15^ Furthermore, to our knowledge, no study has examined the prognostic ability of home pulse rate in patients with hypertension undergoing strict antihypertensive treatment. Based on the multicenter HOMED‐BP (Hypertension Objective Treatment Based on Measurement by Electrical Devices of Blood Pressure) study,^18^ we aim to estimate the prognostic ability of home pulse rate for cardiovascular outcomes.

## METHODS

The data that support the findings of this study are available from the corresponding author upon reasonable request.

### Design

The current study is a post hoc analysis of the HOMED‐BP study,^18^ which was a multicenter trial, PROBE (Prospective, Randomized, Open‐labeled, Blinded Endpoint) with a 2×3 factorial design. The trial compared BP control (tight versus usual) and the drug classes for starting treatment (angiotensin‐converting enzyme inhibitors, angiotensin receptor blockers, or calcium channel blockers). We enrolled the first patient on June 6, 2001, and the last patient on October 7, 2009. The primary outcome of the HOMED‐BP study was assessed as of April 30, 2010, and extended follow‐up observations of BP measurements and outcomes continued until the end of 2012. Antihypertensive medications were adjusted to achieve target home BP levels. First‐line drugs (angiotensin‐converting enzyme inhibitors, angiotensin receptor blockers, or dihydropyridine calcium channel blockers) were started at a lower dose and increased in steps. Diuretics were added in the third step, α‐ or β‐blockers in the fourth, and any antihypertensive agent in the fifth.^19^ The study protocol was approved by the institutional review board of the Teikyo University School of Medicine (17‐044‐3) and conformed to the Helsinki Declaration. Written, informed consent was obtained from all study participants. The HOMED‐BP study was registered with the UMIN Clinical Trial Registry, Number C000000137 (http://www.umin.ac.jp/ctr).

### Study Patients

The HOMED‐BP study enrolled patients with mild‐to‐moderate hypertension, aged 40 to 79 years, who were treated at 457 general practices throughout Japan.^18^ Eligible patients were either treatment‐naïve or previously treated but could discontinue their antihypertensive drug treatment for at least 2 weeks, with a home systolic BP of ≥135 mm Hg or a home diastolic BP of ≥85 mm Hg when off treatment. Patients with any contraindications to antihypertensive agents were excluded. For the present analysis, we pooled data from all 3518 patients assigned to each intervention arm.^20^, ^21^ In the main analysis estimating the prognostic ability of home pulse rate for cardiovascular outcomes, we excluded 374 patients with missing data on home pulse rate during the follow‐up period. Additionally, we excluded 26 patients with atrial fibrillation and 96 patients with a history of cerebrovascular disease at baseline. A total of 3022 patients were analyzed statistically.

### Pulse Rate and Blood Pressure Measurement

Home pulse rate was automatically recorded at the same time as home BP measurement. The HOMED‐BP participants consistently self‐measured every morning throughout the study period, adhering to the Japanese guidelines for home BP monitoring.^9^, ^16^ They measured after resting for ≥2 minutes in a sitting position, within an hour of waking, after urination, before breakfast, and before taking antihypertensive medications. Patients were asked to measure their home pulse rate at least once per occasion. Although they were allowed to take multiple measurements on each occasion, only the first measurement value was used in the analysis in order to eliminate bias from mixing single and multiple measurements. All patients received a validated sphygmomanometer, OMRON HEM 747IC‐N (Omron Healthcare, Kyoto, Japan), which stored readings in its memory.^22^ The device was based on the oscillometric method. The validity of the oscillometric method for measuring pulse rate has previously been reported by Palatini and colleagues.^23^ The baseline home pulse rate and BP were defined as the averages of the 5 consecutive days (5 readings) immediately before randomization (ie, before starting antihypertensive drug treatment). Similarly, the follow‐up home pulse rate and BP were defined as the average of the last available 5‐day home readings (5 readings). However, if patients had an event during the follow‐up period, their follow‐up home pulse rate and BP were the average of the 5‐day readings 6 months before the event.^18^ This 6‐month interval was chosen to minimize bias related to the fall or rise of the follow‐up BP as a precursor to an event.^24^

Health care professionals recorded 2 successive measurements of both pulse rate and BP as the office measurements during each patient visit, after patients had rested for a 2‐minute period in a seated posture. These measurements were taken with the validated oscillometric device, OMRON HEM‐907IT (Omron Healthcare, Kyoto, Japan).^25^ The office pulse rate and BP were determined by averaging these 2 readings.

### Definitions of Events and Covariates

We defined events as major adverse cardiovascular events (MACE) and all‐cause mortality. MACE included stroke (*International Classification of Diseases, Tenth Revision [ICD‐10]* codes: I60, I61, I63), myocardial infarction (I21), and cardiovascular death (I00–I99). We considered only the first event in each patient for analysis. We followed the patients from the baseline home measurement until the end of the follow‐up. We defined diabetes as a fasting plasma glucose level ≥7.0 mmoL/L (126 mg/dL), a glycated hemoglobin level ≥6.5%, or use of antidiabetic agents. We defined hypercholesterolemia as a total cholesterol level ≥5.69 mmoL/L (220 mg/dL), a history of hypercholesterolemia, or use of cholesterol‐lowering medication. We defined anemia as hemoglobin levels <12.0 g/dL in women and <13.0 g/dL in men.

### Statistical Analysis

For testing the linear trend, we used single linear regression analysis for continuous variables and the Cochran‐Armitage trend test for categorical variables. Pearson's correlation coefficients were calculated to determine the correlation between pulse rate values because pulse rates can be treated as a normal distribution. To examine the prognostic ability of home pulse rate, we used Cox proportional hazard models to calculate the hazard ratio and 95% CI for events, with adjustments for age, sex, body mass index, current smoking, current drinking, diabetes, hypercholesterolemia, randomization group, and home systolic BP (the main analysis, model 1, n=3022). We also performed 3 sensitivity analyses. First, we additionally adjusted for the estimated glomerular filtration rate and hemoglobin levels in the patients who had the data available (model 2, n=2869), considering the confounding effects of high pulse rate associated with poor renal function and anemia. Second, we censored events within the first 2 years of follow‐up (model 3, n=3022) to eliminate the increased risk of high pulse rate and mortality associated with the presence of comorbidities. Third, we excluded the patients taking β‐blockers (n=658) because of their profound effect on pulse rate, and then repeated the main analysis in the remaining patients (model 4, n=2364).

We compared the prognostic ability of home pulse rate and office pulse rate using the −2 log likelihood statistic for 2459 patients, excluding those with missing baseline office pulse rate. The −2 log likelihood served as a model fit statistic, with higher values indicating a less accurate model. We compared this statistic among 3 models: the first model, which simultaneously included both home pulse rate and office pulse rate; the second model, derived from the first model by excluding the home pulse rate; and the third model, derived from the first model by excluding the office pulse rate. All models were further adjusted for baseline characteristics including age, sex, body mass index, current smoking, current drinking, diabetes, hypercholesterolemia, randomization group, and home systolic BP. We repeated the analysis at follow‐up (n=2324), excluding those with missing follow‐up office pulse rate.

Subsequently, to propose an outcome‐driven cut‐off point for home pulse rate, we performed receiver‐operating characteristic (ROC) analyses to assess the sensitivity and specificity of home pulse rate measurements in predicting all‐cause mortality. The cut‐off point was set to maximize the Youden index.

Unless otherwise stated, we expressed all data as the means±SDs. We considered a 2‐sided α level <0.05 as significant. We used SAS software, version 9.4 (SAS Institute Inc., Cary, NC), for database management and statistical analysis.

## RESULTS

At baseline, the average home pulse rate was 69.0 (SD, 9.4) beats per minute (bpm), and the range was between 41.6 bpm and 108.6 bpm. The correlation coefficients between the average home pulse rate and the office pulse rate were 0.54 at baseline and 0.53 at follow‐up. Table 1 demonstrates the baseline characteristics of the patients, categorized by quintiles of home pulse rate. The mean age was 59.4 (SD, 9.9) years, and 1518 (50.2%) of the patients were women. Compared with patients in the lowest quintile of home pulse rate, those in the higher quintiles were younger, more likely to be men, smokers, drinkers, and to have diabetes. They were also more likely to be prescribed β‐blockers, and had higher estimated glomerular filtration rate, home diastolic BP, and office diastolic BP.

**Table 1:** Baseline Characteristics of the Patients by Quintiles of Baseline Home Pulse Rate

	Total (n=3022)	Home pulse rate, bpm					*P* value
		41.6–61.1 (n=605)	61.2–66.1 (n=620)	66.2–70.5 (n=584)	70.6–76.2 (n=613)	76.3–108.6 (n=600)	
Age, y	59.4±9.9	62.3±8.7	60.5±9.8	59.7±9.9	58.7±10.1	55.9±9.9	<0.0001
Women, n (%)	1518 (50.2)	312 (51.6)	343 (55.3)	310 (53.1)	313 (51.1)	240 (40.0)	<0.0001
Body mass index ≥25 kg/m^2^, n (%)	1168 (38.6)	220 (36.4)	230 (37.1)	239 (40.9)	243 (39.6)	236 (39.3)	0.1734
Smoking, n (%)	635 (21.0)	98 (16.2)	94 (15.2)	94 (16.1)	128 (20.9)	221 (36.8)	<0.0001
Drinking, n (%)	1463 (48.4)	281 (46.4)	273 (44.0)	275 (47.1)	295 (48.1)	339 (56.5)	0.0002
Diabetes, n (%)	449 (14.9)	72 (11.9)	86 (13.9)	75 (12.8)	97 (15.8)	119 (19.8)	0.0001
Hypercholesterolemia, n (%)	1553 (51.4)	330 (54.5)	318 (51.3)	305 (52.2)	299 (48.8)	301 (50.2)	0.0792
Anemia, n (%)	279 (9.6)	67 (11.4)	56 (9.4)	49 (8.7)	55 (9.3)	52 (9.0)	0.2190
β‐Blockers, n (%)	658 (21.8)	94 (15.5)	120 (19.4)	135 (23.1)	141 (23.0)	168 (28.0)	<0.0001
eGFR, mL/min per 1.73 m^2^	73.1±17.5	70.1±15.4	72.1±16.2	72.9±17.6	73.8±18.5	76.7±19.1	<0.0001
Home pulse rate, bpm	69.0±9.4	56.8±3.4	63.8±1.5	68.3±1.3	73.2±1.6	83.1±6.1	<0.0001
Home systolic BP, mm Hg	151.8±12.5	152.1±12.4	151.7±12.7	151.7±12.6	151.5±12.3	151.9±12.7	0.6772
Home diastolic BP, mm Hg	90.1±10.0	86.3±9.7	88.4±9.5	90.4±9.6	91.5±10.2	93.8±9.6	<0.0001
Office pulse rate, bpm	74.2±13.0	64.5±9.9	70.8±9.9	73.9±11.4	78.0±11.4	84.3±13.2	<0.0001
Office systolic BP, mm Hg	154.4±17.5	155.1±18.1	154.5±17.1	153.3±17.0	153.8±17.5	155.5±17.7	0.7258
Office diastolic BP, mm Hg	90.4±12.2	86.9±12.0	89.0±11.5	90.2±11.4	91.6±12.5	94.4±12.1	<0.0001

bpm indicates beat per minute; and eGFR, estimated glomerular filtration rate. Data on anemia, eGFR, and office measurements were unavailable in 109, 130, and 563 patients, respectively. Diabetes was characterized by a fasting plasma glucose level of 7.0 mmoL/L (126 mg/dL) or higher, a glycated hemoglobin level of 6.5% or higher, or the administration of antidiabetic medications. Hypercholesterolemia was identified by a total cholesterol level of 5.69 mmoL/L (220 mg/dL) or higher, a documented history of hypercholesterolemia, or the use of cholesterol‐lowering drugs. Anemia was defined as hemoglobin levels <12.0 g/dL in women and <13.0 g/dL in men. *P* denotes the significance of the linear trend across categories of home pulse rate.

**Figure 1:**
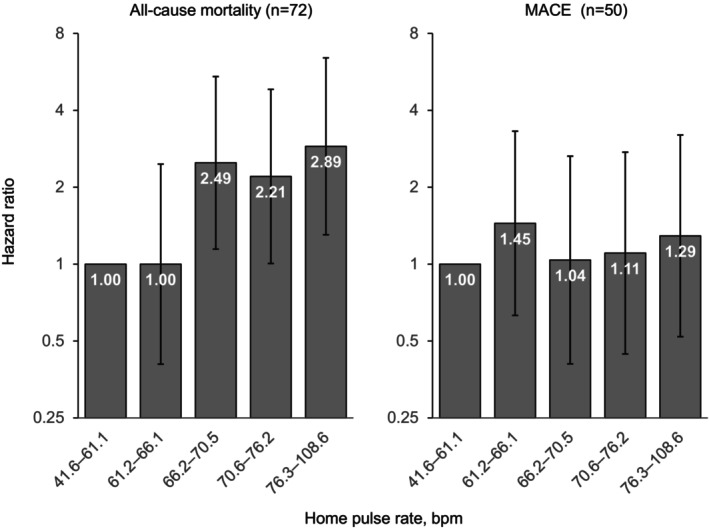
Hazard ratios for all‐cause mortality and MACE by quintiles of baseline home pulse rate at baseline. We adjusted the hazard ratios for sex, age, body mass index, current drinking, smoking, hypercholesterolemia, diabetes, randomization group, and home systolic blood pressure. *MACE was defined as cardiovascular death (*International Classification of Diseases, Tenth Revision* [*ICD‐10*] codes: I00–I99), nonfatal stroke (I60, I61, I63), and nonfatal myocardial infarction (I21). bpm indicates beats per minute; and MACE, major adverse cardiovascular events.

**Figure 2:**
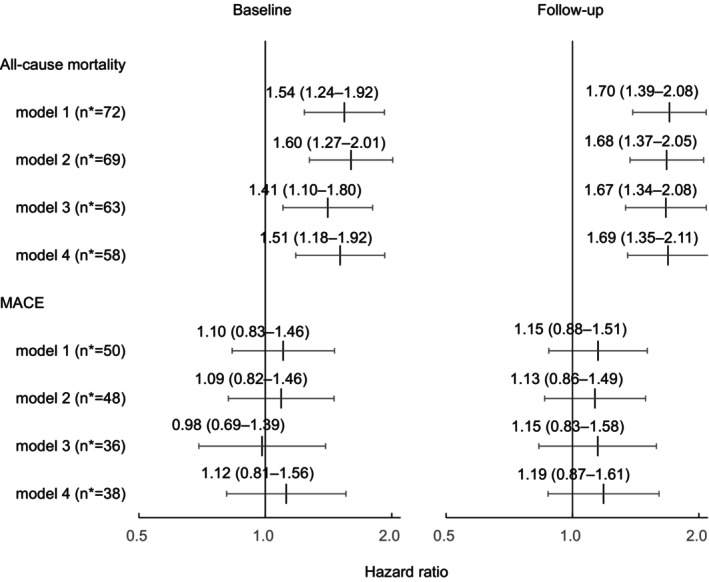
Hazard ratios per 1‐SD increase in home pulse rate for all‐cause mortality and MACE at baseline and follow‐up. *N indicates number of events. We adjusted the hazard ratios for age, sex, body mass index, current smoking, current drinking, diabetes, hypercholesterolemia, randomization group, and home systolic blood pressure (model 1). We also performed 3 sensitivity analyses: further adjustment for estimated glomerular filtration rate and hemoglobin (model 2, number of patients [n]=2869), censoring events within the first 2 years of follow‐up (model 3, [n]=3022), and subgroup analysis in patients without β‐blockers (model 4, [n]=2364). MACE indicates major adverse cardiovascular events.

During a median follow‐up of 7.3 years (interquartile range, 4.8–9.1 years; 20 684 person‐years), we observed 50 MACEs, encompassing 34 strokes, 11 myocardial infarctions, and 5 cardiovascular deaths, and 72 all‐cause deaths. Figure 1 shows the hazard ratios for MACE and all‐cause mortality according to quintiles of baseline pulse rate in model 1. Although no significant results were shown for MACE, the upper 3 quintiles had significantly higher hazard ratios for all‐cause mortality than the lowest quintile. The hazard ratios per 1‐SD increase in home pulse rate at baseline and follow‐up are shown in Figure 2. In model 1, a 1‐SD increase in home pulse rate captured at baseline and follow‐up (ie, 9.4 bpm and 9.9 bpm) corresponded to hazard ratios of 1.54 (95% CI, 1.24–1.92) and 1.70 (95% CI, 1.39–2.08), respectively. A higher pulse rate was significantly and consistently associated with all‐cause mortality among all models. In contrast, no significant association between home pulse rate and MACE was observed (*P* >0.27). Confirmatory results were observed when we assessed the prognostic power of home pulse rate for noncardiovascular deaths (n=66) at baseline (hazard ratio, 1.51 [95% CI, 1.20–1.90]), and at follow‐up (model 1; hazard ratio, 1.69 [95% CI, 1.37–2.08]).

**Figure 3:**
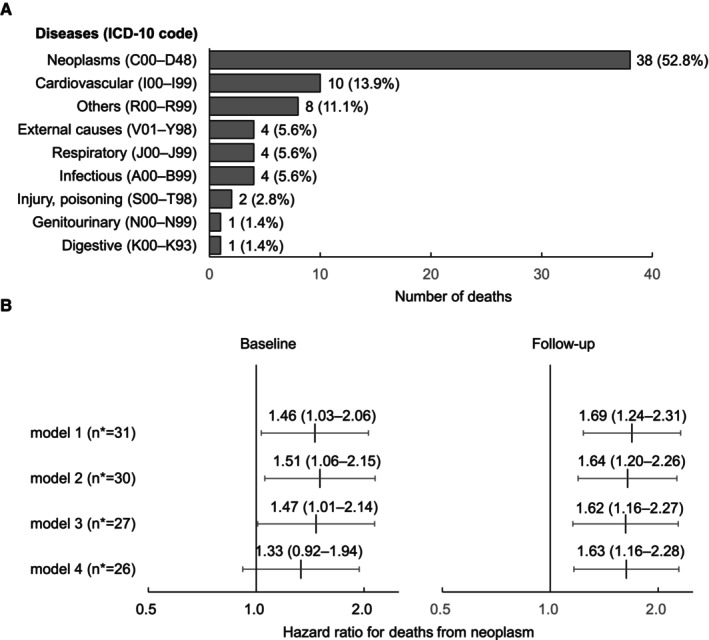
Analysis of cause‐specific deaths and hazard ratios for death from neoplasms. **A**, Shows the number and percentage of deaths classified by codes from the *International Classification of Diseases, Tenth Revision* (*ICD‐10* code). Among the 72 deaths, more than half were due to neoplasms (n=38). (**B**) Shows the hazard ratios for death from neoplasms per 1‐SD increase in home pulse rate at baseline and follow‐up. *N indicates number of events. We adjusted the hazard ratios for age, sex, body mass index, current smoking, current drinking, diabetes, hypercholesterolemia, randomization group, and home systolic blood pressure (model 1). We also performed 3 sensitivity analyses: further adjustment for estimated glomerular filtration rate and hemoglobin (model 2, number of patients [n]=2869), censoring events within the first 2 years of follow‐up (model 3, n=3022), and subgroup analysis in patients without β‐blockers (model 4, n=2364).

As shown in Figure 3, more than half (n=38) of the 72 deaths were due to neoplasms (upper panel). Similar to the risk of all‐cause mortality, we observed significantly elevated hazard ratios associated with home pulse rate, irrespective of baseline or follow‐up measurements, for deaths related to neoplasms. The only exception was a nonsignificant hazard ratio in model 4 at baseline (*P*=0.13) as shown in Figure 3B.

We compared the prognostic ability of the home pulse rate and office pulse rate (Table 2). In a model that simultaneously included baseline home pulse rate and office pulse rate, only the home pulse rate significantly predicted all‐cause mortality (*P*=0.019), not the office pulse rate. When we removed the home pulse rate from this model, the −2 log likelihood statistic significantly increased from 798.9 to 804.2 (*P*=0.020). Conversely, the model from which the office pulse rate was removed, instead of the home pulse rate, showed almost no change in the −2 log likelihood statistic. Similar results were obtained for follow‐up pulse measurements.

**Table 2:** Comparison of Model Fit Between a Cox Model for All‐Cause Mortality That Includes Both Home Pulse Rate and Office Pulse Rate and a Model That Excludes One of Each

Period of pulse rate measurement (number of events/patient)	Both home pulse rate and office pulse rate were simultaneously included in the same model					Removing home pulse rate	Removing office pulse rate
	Home pulse rate		Office pulse rate		–2 Log likelihood	–2 Log likelihood	–2 Log likelihood
	hazard ratio (95% CI)	*P* value	hazard ratio (95% CI)	*P* value			
Baseline (58/2459)	1.42 (1.06–1.91)	0.019	1.04 (0.77–1.39)	0.810	798.9	804.2*	798.9
Follow‐up (52/2324)	1.50 (1.12–2.01)	0.007	1.16 (0.87–1.56)	0.320	706.6	713.3*	707.6

The −2 log likelihood served as a model fit statistic, with higher values indicating a less accurate model. We compared this statistic among 3 models: the first model, which simultaneously included both home pulse rate and office pulse rate; the second model, which was derived from the first model by removing the home pulse rate; and the third model, which was derived from the first model by removing the office pulse rate. All models were further adjusted for baseline characteristics including age, sex, body mass index, current smoking, current drinking, diabetes, hypercholesterolemia, randomization group, and home systolic blood pressure.

* *P*=0.020 for baseline and *P*=0.009 for follow‐up, compared with a model that simultaneously includes both home pulse rate and office pulse rate.

**Figure 4:**
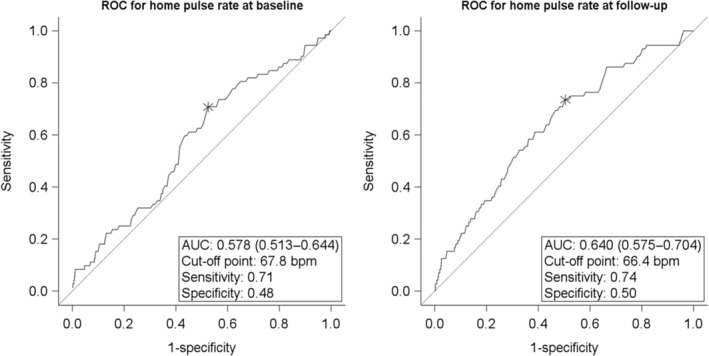
ROC analyses demonstrating the optimal cut‐off for home pulse rate in predicting all‐cause mortality. AUC represents the area under the curve with its 95% CI; bpm, beats per minute; and ROC receiver‐operating characteristic.

Based on the receiver‐operating characteristic analyses (Figure 4), the area under the curve of home pulse rate for predicting all‐cause mortality was 0.578 (0.513–0.644) at baseline and 0.640 (0.575–0.704) during follow‐up. The corresponding cut‐off points were 67.8 bpm, with the sensitivity of 0.71 and specificity of 0.48, and 66.4 bpm, with a sensitivity of 0.74 and a specificity of 0.50, respectively.

## DISCUSSION

Our study revealed an association between home pulse rate, both before and during antihypertensive treatment, and the risk of all‐cause, noncardiovascular, and neoplasm mortality. There may exist untapped potential for further prognostic refinement even among patients undergoing rigorous antihypertensive treatment. This finding underscores the need for research into treatment strategies that leverage home pulse rate as a vital indicator. Our findings also highlighted the superior prognostic value of the home pulse rate in comparison to the office pulse rate.

Although numerous studies^1^, ^2^, ^3^, ^4^, ^5^, ^6^, ^7^ have examined the relationship between office pulse rate and hard outcomes, there are few studies on out‐of‐office pulse rate,^12^, ^13^, ^14^, ^15^ particularly on home pulse rate.^15^ Our group previously reported^15^ that among 1780 general residents aged 40 years or older in Ohasama, Japan, those with a home pulse rate in the 4th quintile or higher (threshold ≥70 bpm) had a significantly higher risk of cardiovascular mortality. The hazard ratios for the 4th and 5th quintiles were 2.54 (95% CI, 1.16–5.58) and 2.61 (95% CI, 1.29–5.31), respectively. In our present analysis of the HOMED‐BP study, home pulse rate was significantly associated with all‐cause mortality from the 3rd quintile or higher group (threshold ≥66.2 bpm) at baseline. The cut‐off obtained by receiver‐operating characteristic analysis of home pulse was 67.8 bpm at baseline and was similar during follow‐up (66.4 bpm). Although the type of events differs, the outcome‐driven threshold for home pulse rate is likely to be 65 to 70 bpm.

In 2293 patients with hypertension assigned to the placebo arm in the Systolic Hypertension in Europe Trial,^7^ office pulse rate was significantly associated with total mortality, and 24‐hour ambulatory pulse rate predicted noncardiovascular mortality. A study by Benetos et al.^3^ also reported that conventional pulse rate was an independent predictor of noncardiovascular mortality in a French population. In a cohort study of 92 562 Chinese community residents,^4^ pulse rate predicted all‐cause mortality (n=1589) but not cardiovascular disease events (n=1903). In the Japanese general population,^12^ the nighttime pulse rate was associated with all‐cause mortality. These results were consistent with the present HOMED‐BP study, although the timing and method of measurements and the target population differed. Meanwhile, Wang and colleagues reported inconsistent results (ie, resting heart rate captured by electrocardiography predicted myocardial infarction and all‐cause mortality but not all‐cause cardiovascular events and stroke).^4^ A meta‐analysis from the Asia Pacific Cohort Studies Collaboration^5^ reported that resting heart rate had positive associations with total and cardiovascular mortality, and fatal and nonfatal cardiovascular disease, coronary heart disease, and stroke when above a threshold of 65 bpm in 112 680 participants from 12 cohort studies. The present study also showed a significantly increased risk of all‐cause mortality in patients with a heart rate ≥66.2 bpm, which was the threshold of the 3rd quartile in Figure 1. However, we did not find any association between pulse rate and MACE. One possible reason was that antihypertensive treatment may have attenuated the impact of pulse rate in well‐managed patients with hypertension. More than half of the HOMED‐BP patients (55.8%) achieved a systolic home BP <130 mm Hg and thereby reduced their risk of a primary cardiovascular end point to 1% or less.^18^ It is likely that the relationship between pulse rate and MACE was not detected in the HOMED‐BP patients because cardiovascular events were less frequent due to adequate BP control.

In the present study, death from neoplasms, which was a leading cause of mortality, had a strong association with heart rate. Gann et al^26^ reported that heart rate determined by an ECG predicted prostate cancer mortality in 22 380 men enrolled in the Chicago Heart Association cohort. A high heart rate was also associated with an increased risk of overall cancer mortality in 50 108 patients from the Cooper Clinic in Dallas, Texas.^27^ A meta‐analysis^28^ including 12 studies showed that the summary relative risk and 95% CIs per 10 bpm increase in resting heart rate was 1.14 (95% CI, 1.06–1.23; I^2^=90.2%) for total cancer. The mechanism underlying the relationship between heart rate and cancer is unknown; however, 1 hypothesis is that an increased heart rate indicates sympathetic nervous activity, and increased sympathetic nerve activity may be involved in cancer progression. Renz et al^29^ uncovered a relationship between the expression of brain‐derived neurotrophic factor, nerve density, and increased survival of patients on nonselective β‐blockers in the cohort of patients with pancreatic ductal adenocarcinoma. Kamiya et al^30^ reported that the autonomic nervous system invades breast cancer tissue as breast cancer increases, strongly influencing tumor progression and prognosis.

These findings suggest that the activation of sympathetic nerves could be associated with the initiation and progression of cancer. The observed association between a high pulse rate and an increased risk of cancer mortality in this study may be attributed to the pulse rate serving as an indicator of sympathetic nerve activation. As a mechanism for the association between pulse rate and cancer mortality, reverse causation must also be considered as a plausible hypothesis. Symptoms associated with tumors, such as pain, bleeding, fatigue, or respiratory symptoms, may cause an increase in pulse rate. However, the adjustment for hemoglobin levels as a confounder in the sensitivity analysis did not substantially affect the main results but may not have sufficiently excluded the effect of tumor‐associated anemia on pulse rate, because there are individual differences in the relationship among hemoglobin levels, anemia symptoms, and tachycardia.

Modifiable lifestyle changes, such as altering smoking habits or leading a less sedentary lifestyle, may improve patient prognosis and aid in reducing pulse rate. In hypertensive patients undergoing antihypertensive treatment, factors such as younger age, diabetes, habitual smoking, high diastolic BP, and not using a β‐blocker or angiotensin‐converting enzyme inhibitor were determinants of home pulse rate elevation.^31^ In the general population of the town of Ohasama, home pulse rate was associated with women, younger age, smoking, sedentary lifestyle, and the white coat effect.^32^ Meanwhile, even though β‐blockers are a common agent for reducing pulse rate, they have a significantly lower preventive effect against stroke and all‐cause mortality compared with other antihypertensive drugs.^33^ This suggests that the primary goal for patients with hypertension is not merely lowering the pulse rate.

With advances in recent technology, wearable devices can provide a means of obtaining pulse rate data outside the clinical setting.^34^, ^35^ They are reported to be particularly effective in detecting atrial fibrillation.^35^ However, their prognostic value with regard to mortality or cardiovascular events has not yet been reported. Future research should compare the findings of this study based on the validated oscillometric device^22^, ^23^ with data obtained from wearable devices.

This study is the first to evaluate and compare the prognostic capabilities of home and office pulse rates. Our findings suggest that the home pulse rate significantly contributes to the predictive power for all‐cause mortality, even in models that already incorporate the office pulse rate. In contrast, office pulse rate does not offer additional prognostic value in models that include home pulse rate. These results indicate that the home pulse rate has superior prognostic power compared with the office pulse rate. However, related evidence is still limited, and further investigations with other populations are warranted. It is also crucial to explore why home pulse rates exhibit better prognostic ability than office pulse rates. The superior predictive power of home pulse rates over office pulse rates could be inferred by drawing parallels with the reasons why out‐of‐office BP has better prognostic ability than office BP. The advantages associated with out‐of‐office BP measurements, such as the absence of alarm response, improved reproducibility, and a larger number of measurements,^9^, ^16^ might also be applicable to home pulse rates compared with office pulse rates. Given the lack of research examining the characteristics of pulse rates, further validation is needed as a future research topic. Currently, the majority of Japanese people routinely measure home pulse rates,^17^ and it is imperative that we utilize this information effectively. Therefore, there is a pressing need for further investigation to ensure that home pulse rate data can be appropriately utilized by clinicians.

This study has some limitations. First, this is a post hoc analysis, and we cannot infer a causal relationship between lowering pulse rate and decreasing mortality rates even though β‐blockers and other antihypertensive agents could amend pulse rate. The rate of β‐blocker use was higher in the higher home pulse rate quintiles (Table 1), suggesting the effect of reverse causality (ie, clinicians might prescribe β‐blockers to try to reduce the pulse rate). A randomized controlled trial that primarily aims to reduce home pulse rate is needed to clarify the causal relationship. Second, data on potential confounders of pulse rate (eg, physical activity or respiratory function) are lacking. Third, the number of study participants reduced from the original 3518 HOMED‐BP population due to missing data, which may result in selection bias.

## CONCLUSIONS

In patients with mild‐to‐moderate hypertension, pulse rates self‐measured at home before antihypertensive drug initiation and during antihypertensive treatment were not associated with the risk of MACE but were associated with the risk of death. Home pulse rate demonstrated superior prognostic ability for mortality compared with office pulse rate. The cut‐off point for home pulse rate based on the receiver‐operating characteristic analysis was 66.2 to 67.8 bpm in the present study. The accuracy of risk stratification can be improved by using home pulse rate, which is easily and frequently self‐measured at home. In the real‐world setting, a clinician should recognize that a patient with a high pulse rate would be at an elevated mortality risk. Identifying, improving, or treating modifiable lifestyle habits and concomitant diseases that may be present alongside the high pulse rate, such as habitual smoking, sedentary lifestyle, diabetes, respiratory disease, or cancer, would be also crucial in clinical practice.

Our findings indicate that further research into the prognostic significance of out‐of‐office pulse rate is warranted. Specifically, pulse rate measurement using wearable devices, which was not included in this study, could have significant clinical and public health implications due to their growing popularity.^36^ Continued development in this area is demanded.

## Sources of Funding

This study was funded by grants from the Japan Cardiovascular Research Foundation, the Japan Arteriosclerosis Prevention Fund, and Grants‐in‐Aid for Scientific Research from the Japan Society for the Promotion of Science (21H04854, 21H04984, 21K19670, 22H03353, 22H03358, 23H03165, 23K24616). This study was also supported by Advanced Comprehensive Research Organization Incubation Grants of Teikyo University. The funding agencies had no role in the study design, data collection, analysis, or interpretation, or the writing, review, or approval of the manuscript.

## Disclosures

Drs Asayama, Imai, and Ohkubo were concurrent directors of the Tohoku Institute for Management of BP, which was supported by Omron Healthcare Co., Ltd. The remaining authors have no disclosures to report.
